# The Role of Triplet States in the Photodissociation of a Platinum Azide Complex by a Density Matrix Renormalization Group Method

**DOI:** 10.1021/acs.jpclett.1c00829

**Published:** 2021-05-18

**Authors:** Leon Freitag, Leticia González

**Affiliations:** Institute for Theoretical Chemistry, Faculty of Chemistry, University of Vienna, Währinger Straße 17, 1090 Vienna, Austria

## Abstract

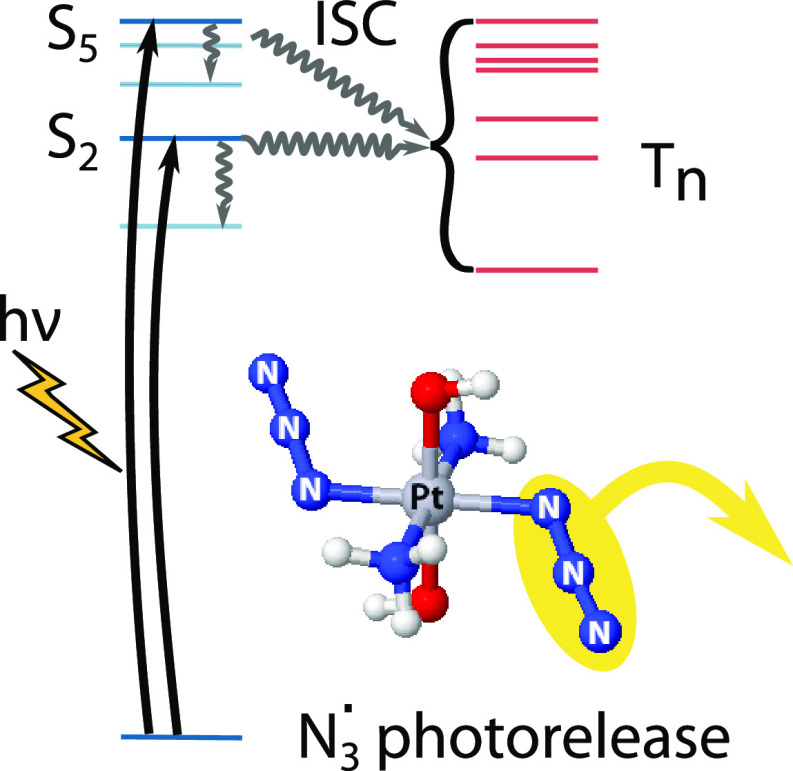

Platinum azide complexes are appealing anticancer photochemotherapy drug candidates because they release cytotoxic azide radicals upon light irradiation. Here we present a density matrix renormalization group self-consistent field (DMRG-SCF) study of the azide photodissociation mechanism of *trans,trans,trans*-[Pt(N_3_)_2_(OH)_2_(NH_3_)_2_], including spin–orbit coupling. We find a complex interplay of singlet and triplet electronic excited states that falls into three different dissociation channels at well-separated energies. These channels can be accessed either via direct excitation into barrierless dissociative states or via intermediate doorway states from which the system undergoes non-radiative internal conversion and intersystem crossing. The high density of states, particularly of spin-mixed states, is key to aid non-radiative population transfer and enhance photodissociation along the lowest electronic excited states.

Platinum-based compounds have been successfully employed in chemotherapy against cancer since the 1970s. However, chemotherapy often shows poor selectivity against tumor cells, leading to poor efficacy and severe side effects. Selectivity, at least for locally confined tumors, can be retrieved using photoactivated chemotherapy,^1^ where anticancer agents are activated with light in a particular body area. Another advantage of photoactivated chemotherapy is that it does not require photosensitizers or the presence of oxygen in the cell, and thus it does not suffer from limited efficacy in hypoxic tumors^2^ or increased patients’ sensitivity to light,^3^ in contrast to older and more established photodynamic therapy.^3−7^

Platinum(IV) complexes,^8−17^ and in particular Pt(IV) azides,^10,18−26^ count among the first and yet most promising photoactivated drug candidates because they are stable in the absence of light and only upon light irradiation release highly cytotoxic products.^26−28^ Accordingly, their photoreaction pathways have been heavily investigated experimentally^10,18,27−34^ but less so computationally.^35−38^ Thus, understanding the major photoreaction pathway at the atomic level—i.e., the dissociation of the azide ligands that recombine to nitrogen along with the reduction of the metal center to Pt(II)^17^—remains elusive.

Due to its moderate computational cost, density functional theory (DFT) and its time-dependent version (TD-DFT) have been the preferred electronic structure methods for computational studies of the photochemistry of transition metal complexes.^39^ Previous computational studies on the dissociation mechanism of Pt(IV) azides employed DFT and TD-DFT to calculate excited-state energies and orbital characters of the equilibrium structure, as well as to perform excited-state structure optimizations^35,37,40^ and potential energy surface scans^35^ that identify dissociative states and explore possible dissociation pathways. However, DFT can be problematic to describe dissociation,^41^ where large amounts of non-dynamical correlation are needed. In these cases, a multiconfigurational method is mandatory^42^ but often prohibitive, especially in transition metal complexes where large active orbital spaces are necessary.^43^ Density matrix renormalization group (DMRG)-based methods,^43−47^ including that with self-consistent field (DMRG-SCF),^48,49^ are ideally suited for multiconfigurational studies of transition metal complexes, as they allow for significantly larger active orbital spaces than traditional multiconfigurational methods. On top of that, DMRG calculations combined with entanglement analysis^50,51^ can be employed for an automated selection of active orbitals,^52,53^ easing the applicability of multiconfigurational methods. Here one should mention that Sokolov and Schaefer^37^ already employed a multiconfigurational method, namely the complete active space self-consistent field/second-order perturbation theory (CASSCF/CASPT2), but not to describe dissociation.

In this work we present the first study of the early stages of azide photodissociation in the flagship Pt(IV) azide complex, *trans,trans,trans*-[Pt(N_3_)_2_(OH)_2_(NH_3_)_2_] (**1** in Scheme 1) with DMRG-SCF. Being a multiconfigurational method, DMRG-SCF allows going beyond the single-configurational framework of DFT employed until now for such complexes. Moreover, for the first time, the role of not only singlet states but also triplet states is investigated, including also spin–orbit interactions. This allows us to unravel the full complexity of azide dissociation pathways.

**Scheme 1:**
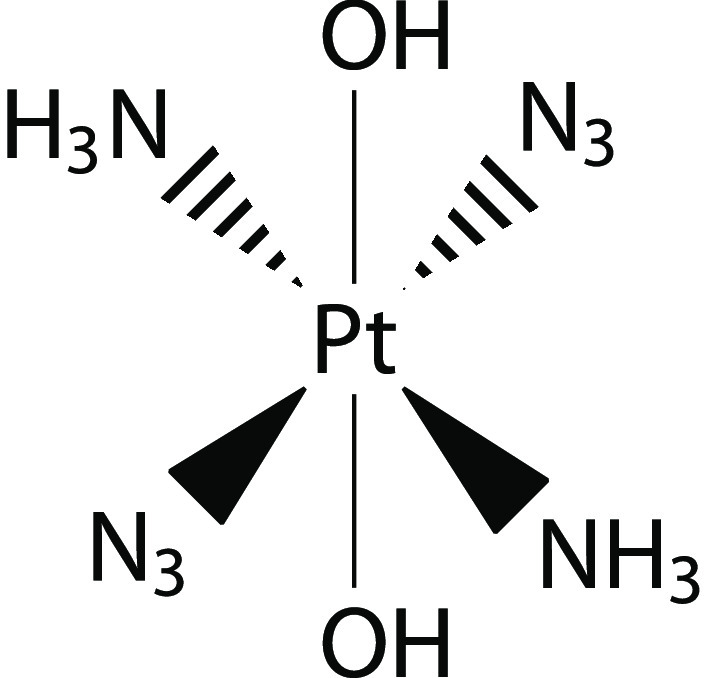
Complex **1**, *trans,trans,trans*-[Pt(N_3_)_2_(OH)_2_(NH_3_)_2_], Studied in This Work

We have identified a relevant active space of 26 electrons in 19 orbitals, comprising azide π bonding, non-bonding, and π* antibonding orbitals, six for each azide ligand (12 in total), five Pt 5*d* orbitals, and additionally two σ orbitals forming bonding–antibonding pairs with the vacant Pt *d* orbitals (see Figure S1 and further details in the Supporting Information (SI)). Using DMRG(26,19)-SCF, we first investigate the nature of the electronic excited states responsible of the absorption spectrum (Figure 1). The obtained Gaussian-convoluted spectrum shows a reasonable qualitative agreement with the experimental one in water, with an overestimation of the main peak by 0.6 eV. Quantitative agreement can be expected upon improving the inclusion of dynamical correlation with perturbation theory and the treatment of solvent effect. Exemplary multireference perturbation theory calculations in the gas phase show that the overestimation of the excitation energies of the lowest two singlet states decreases by 0.23–0.30 eV, thus improving the agreement with the experiment (see more details in the SI). We expect a similar effect for higher electronic excitations.

**Figure 1:**
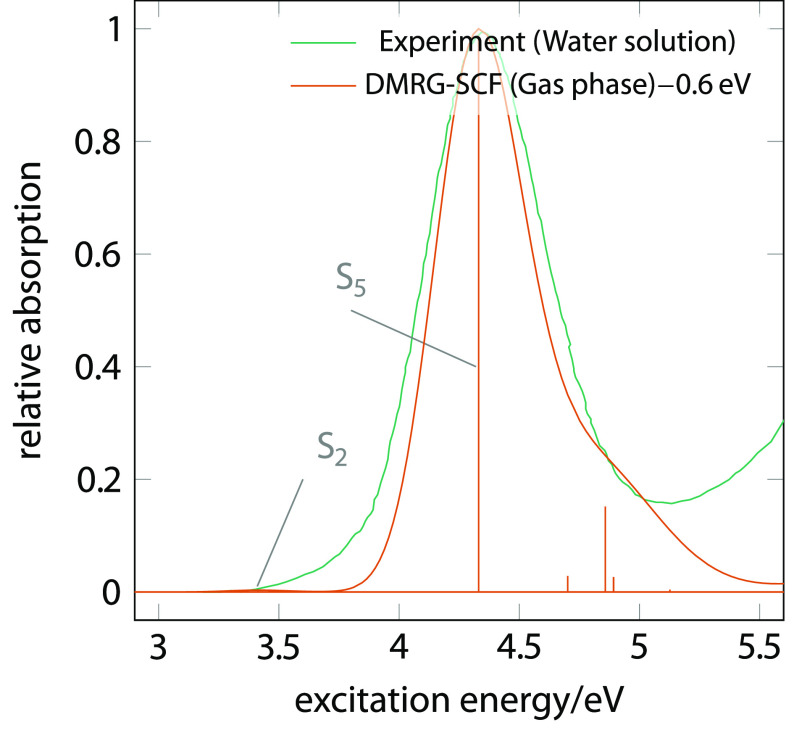
Convoluted DMRG-SCF absorption spectrum of **1** (red-shifted by 0.6 eV), along with the assignments of the peaks to the important bright states and the experimental^20^ absorption spectrum.

A comprehensive overview of the lowest singlet and triplet excitation energies and state characters can be found in Table S1 in the SI. The absorption maximum is attributed to the bright S_5_ state. The S_2_ state, with an oscillator strength ca. 300 times smaller than that of S_5_, can explain the photoreactivity of **1** observed at energies lower than the absorption maximum.^27^ A similar reactivity has also been observed in the *cis* analog of **1**.^19^

As can be seen from the natural transition orbitals (cf. Figure 2), the S_1_, S_2_, and S_5_ states consist of excitations to the Pt 5*d*_*z*^2^_ orbital, forming an antibonding σ* linear combination with an azide π orbital, and thus are expected to feature dissociative character. In contrast, the other two lowest singlet states, S_3_ and S_4_, correspond to *d*→*d* excitations into the Pt 5*d*_*x*^2^–*y*^2^_ orbital and hence should not be dissociative. We note that, in the *cis* analog of **1**, all four lowest-lying excited states have been proposed^35^ to be dissociative, but that is based on TD-DFT calculations.

**Figure 2:**
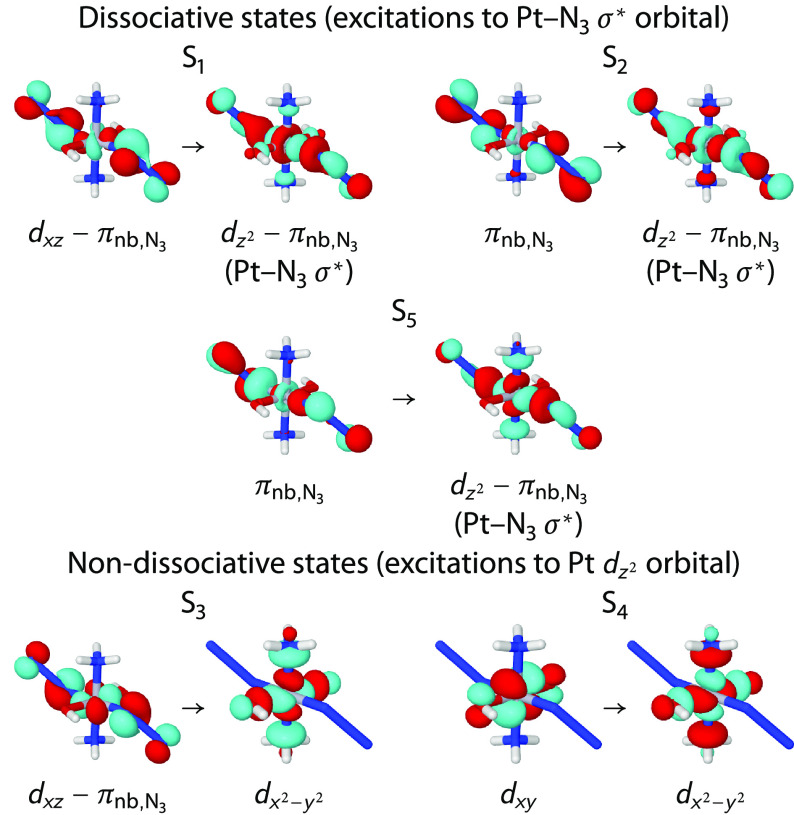
Natural transition orbitals for the first five singlet excited states in complex **1**.

Some of the calculated triplet states (see Table S1) are also identified as dissociative. As expected, T_1_ has a similar character as the S_1_. Also the T_2_, T_5_, and T_6_ states can be assigned as excitations into the Pt–N_3_ antibonding orbitals. The large number of low-lying states with dissociative character suggests that many direct dissociation channels are available after the complex is photoexcited to the S_5_ or S_2_ states.

In order to gain a deeper understanding of the photodissociation mechanism of **1**, it is mandatory to move away from the Franck–Condon geometry. Accordingly, we calculated unrelaxed potential energy curves of the lowest 11 singlet and 9 triplet (spin-free) states along the Pt–N_3_ bond (see Figure 3). As hypothesized, several states of both multiplicities show barrierless dissociative profiles, which should ease a single azide ligand dissociation. The depicted states give access to three asymptotic dissociation channels, namely (i) at around 2 eV, arising from the S_0_, S_1_, T_1_, and T_2_ states; (ii) at 4 eV, from the S_5_ (via S_4_ and S_3_) and T_9_ states (via lower triplet states); and (iii) above 5 eV, coming from higher-lying singlet and triplet states. Upon excitation into the lowest-lying absorption maximum, i.e. S_5_, two out of the three channels—at 2 and 4 eV—are energetically available, while the excitation into S_2_ can only access the dissociation channel at 2 eV—access to the 4 eV channel is not barrierless. If we consider that molecules will quickly deactivate non-radiatively to the lowest electronic state (Kasha–Vavilov rule^54^), dissociation is expected to take place ultimately from the 2 eV channel.

**Figure 3:**
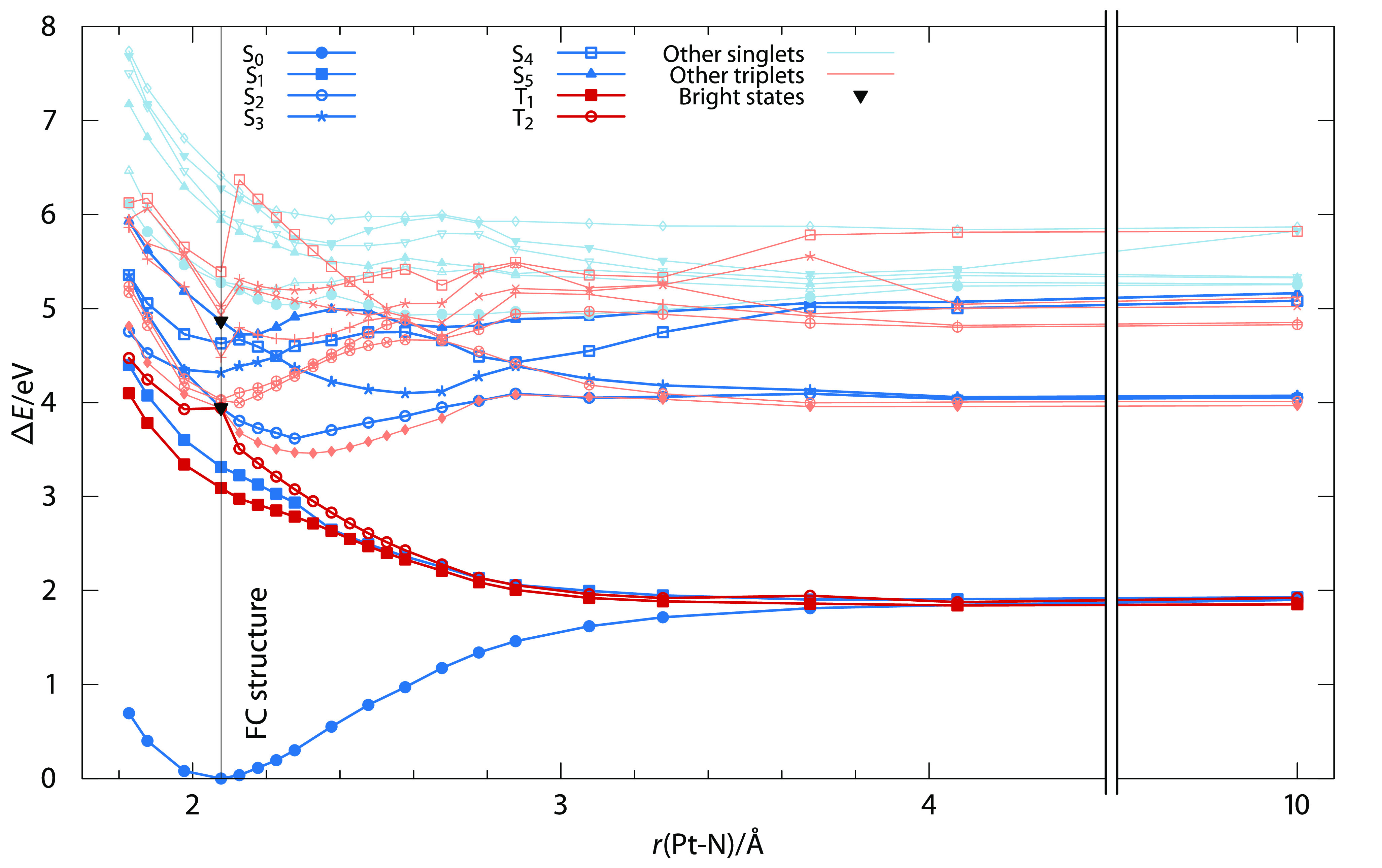
Spin-free potential energy curves of 11 singlet and 9 triplet states along the Pt–N_3_ bond in the Pt complex. Bright states at the equilibrium bond Pt–N_3_ length of 2.077 Å (marked with a straight line) are highlighted with an inverted triangle. Darker colors indicate the most relevant states discussed in the text.

In this spin-free representation, where spin–orbit-coupling is neglected, the channels at 2 and 4 eV each consist of four-fold degenerate states—two singlet and two triplet states. The degeneracy of the two states in each spin multiplicity arises due to symmetry—they share one of the two singly occupied molecular orbitals (SOMOs), while the other SOMOs are orthogonal π non-bonding orbitals of the azide ligands (in Figure 4: (a) and (b) for the 2 eV dissociation channel, (b) and (d) for the 4 eV channel). For the 2 eV channel, the shared SOMO is a mixture of *d*_*z*^2^_ and non-bonding azide π orbital with an antibonding interaction (Figure 4c), while in the 4 eV channel, it is a pure azide π non-bonding orbital (Figure 4a).

**Figure 4:**
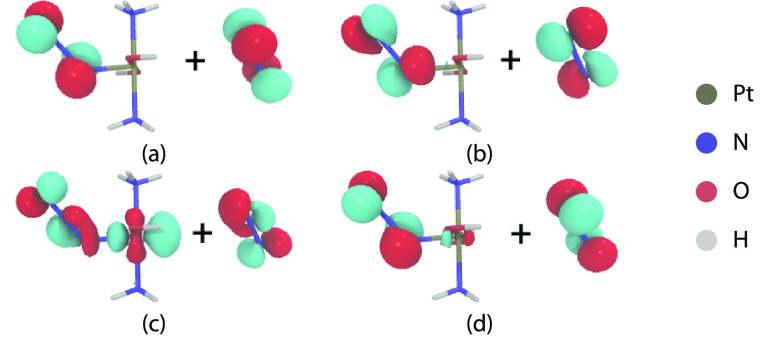
Shape of the different singly occupied molecular orbitals that represent the degenerate states at the 2 and 4 eV dissociation channels. The two singly occupied orbitals for both S_0_ and T_1_ are (b) and (c), for S_1_ and T_2_ (a) and (c), and in the 4 eV channel for S_2_ and T_3_ (a) and (b), for S_3_ and T_4_ (a) and (d).

The open-shell character of the azide singly occupied orbital in all states of both dissociation channels confirms that the azide dissociates as a radical. The singly occupied orbital (with a metal contribution) in the 2 eV channel shows a σ* antibonding interaction that weakens the Pt–N_3_ bond of the undissociated azide ligand, hinting at an easy dissociation of the second azide from this channel.

The high density of excited states is expected to contribute to the rapid and efficient photocleavage. This is particularly true around the equilibrium structure, where the S_5_ state lies closely to two other singlet and three triplet states, allowing for a rapid population transfer via both internal conversion and intersystem crossing. Although those states are not dissociative themselves, they act as intermediate doorway states to promote non-radiative deactivation toward the dissociation channels.

Spin–orbit couplings induce a large energy splitting of the spin-free states at and near the equilibrium structure. As can be seen from the first column in Figure 5, the spin–orbit states have a significantly larger density of states than the spin-free states across the whole energy range up to 5.5 eV. The increase of the density of states is expected to enhance the efficacy of the deactivation toward the dissociation channels. With spin–orbit couplings of up to 1800 cm^–1^ between singlet and triplet excited states, ultrafast intersystem crossing is possible shortly after photoexcitation: in fact, in the spin–orbit representation, both bright S_2_ and S_5_ states show a significant amount of triplet character and significant spin–orbit interaction with close-lying triplet states. With increasing Pt–N bond length, spin–orbit couplings remain large, and thus spin-mixed states abound. Although in the upper energy range the spin–orbit splittings remain large, this is not the case for the lower-energy excited states, which collapse to the 2 and 4 eV dissociation channels: here the spin–orbit energy splitting becomes less pronounced and approaches zero toward the dissociation limit, allowing for the same picture of two dissociation channels as in the spin–orbit picture (see middle and last columns in Figure 5).

**Figure 5:**
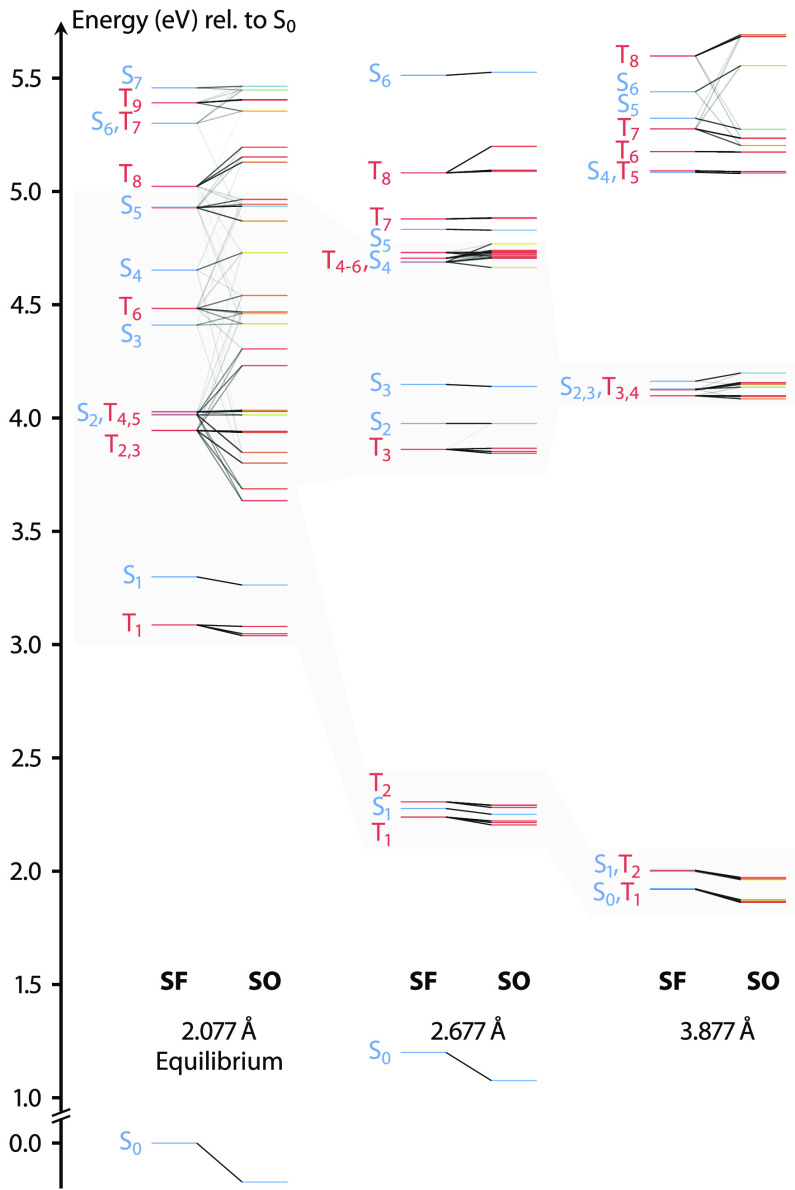
Spin–orbit splitting of the state energies and characters at equilibrium structure and selected Pt–N distances. For each Pt–N distance, the spin-free (SF) and spin–orbit (SO) energies are shown in the left and right columns, respectively. SF and pure singlet and triplet states are denoted with blue and red, and SO mixed states with shades of yellow and green. The states available for deactivation after the excitation at the absorption maximum and the dissociation channels at 2 and 4 eV are highlighted in gray.

Accordingly, spin–orbit coupling plays a significant role in funneling the population toward the dissociation channels shortly after photoexcitation but is less important at later stages.

The obtained results allow us then to summarize the photodissociation mechanism of the Pt complex **1** as follows. Absorption near the maximum at 4.93 eV (252 nm) excites the molecule into the S_5_ state. This state has dissociative character, as shown in the natural transition orbitals at the equilibrium structure and the topology of its potential energy curve along the Pt–N bond; thus the S_5_ excitation may lead the molecule, via avoided crossings, to S_4_ and S_3_ close to the Franck–Condon structure and to S_2_ at approximately 2.7 Å directly into the dissociation channel around 4 eV. Internal conversion and intersystem crossing are expected to compete to transfer non-adiabatically the population to lower-lying excited singlet and triplet states, which ultimately opens the dissociation channel at around 2 eV, consisting of S_0_, S_1_, T_1_, and T_2_ states. Although some of these lower-lying excited states are not dissociative, they still serve as a doorway to the dissociative lower states. Large spin–orbit couplings should facilitate ultrafast intersystem crossing on one hand and increase the density of states close to the equilibrium structure on the other hand, favoring population transfer toward lower-lying dissociative states. By virtue of intersystem crossing, dissociation in the triplet states should be open along the upper dissociation channel at 4 eV. Upon excitation to S_2_, deactivation via internal conversion and intersystem crossing will dissociate the azide along the 2 eV dissociation channel.

The present work thus offers a comprehensive mechanism for the dissociation of a Pt azide complex and highlights the pivotal role of the triplet states and the spin–orbit couplings. Further mechanistic implications of these non-adiabatic processes remain an eventual target for dynamical simulations. We have shown the versatility of DMRG-SCF to describe dissociation accurately, overcoming the limitations of density functional theory. In a broader context, this study demonstrates the very general applicability of DMRG-SCF calculations with active spaces not accessible by traditional multiconfigurational complete active space-based methods, paving the way to elucidate further photodissociation mechanisms in transition metal complexes with entangled quantum many-electron calculations.

## Supporting Information Available

Computational details, active space orbitals, spin-free and spin–orbit energies at the equilibrium structure, and spin–orbit couplings (PDF)
