# Supplementary material for: The Role of Triplet States in the Photodissociation of a Platinum Azide Complex by a Density Matrix Renormalization Group Method
Source: J Phys Chem Lett. 2021 May 18;12(20):4876–81. doi: 10.1021/acs.jpclett.1c00829 (PMC8165699; doi:10.1021/acs.jpclett.1c00829)
Supplement: Supplementary file 1 — jz1c00829_si_001.pdf [file jz1c00829_si_001.pdf]

Supporting information for: The Role of Triplet States in the Photodissociation of a Platinum Azide Complex by a Density Matrix Renormalization Group Method

Leon Freitag* and Leticia González*

Institute for Theoretical Chemistry, Faculty of Chemistry, University of Vienna, Währinger Str. 17, 1090 Vienna, Austria

E-mail: leon.freitag@univie.ac.at; leticia.gonzalez@univie.ac.at

Computational Details

The equilibrium gas-phase molecular structure of the complex has been optimised with density functional theory (DFT), employing the BP86 functional,[1,2] the Ahlrich's def2-TZVPP basis set[3–5] and Grimme's D3 dispersion correction.[6] Except in pathological cases, DFT is commonly used to optimise equilibrium structures of transition metal complexes, which then are used in subsequent ab-initio (multiconfigurational) computational studies, see e. g. Refs. 7 and 8. In particular, DFT has shown to perform well for the optimisation of the equilibrium structures of Pt(IV) azido complexes.[9] To account for the scalar relativistic effects in Pt, the relativistic 60-electron effective core potential (MWB60 ECP) has been used.[10] These calculation have been performed with the TURBOMOLE 7.0[11] program package.

Using the above molecular structure, multiconfigurational density matrix renormalisation group-self consistent field (DMRG-SCF)[12,13] calculations have been carried out with an active orbital space consisting of 26 electrons in 19 orbitals. The comprising orbitals are depicted in Fig. S1. The number of renormalised block states (m) has been set to 500. To estimate the effect of m on the accuracy of the calculation, we also performed a single-point calculation of singlet states with $m = 1000$ at the equilibrium structure, but the excitation energies differed only slightly from those obtained with $m = 500$ (see Table S3): hence, for performance reasons we settled for a value of $m = 500$ for the remaining calculations. To validate the results further, we performed multiconfigurational calculations that include dynamic correlation, namely quasi-degenerate n-electron valence based second-order perturbation theory calculations based on our DMRG-SCF reference wavefunction with $m = 500$ (DMRG-QD-NEVPT2).[14–17] Due to a very large computational cost, only three lowest singlet state energies at the Franck-Condon structure were computed. The results are presented in Table S4.

These calculations employed the all-electron ANO-RCC valence quadruple-zeta polarised (ANO-RCC-VQZP) basis set[18] for Pt and its more compact triple-zeta pendant, ANO-RCC-VTZP, for remaining atoms. Two-electron integrals were calculated with the atomic compact

Cholesky decomposition (CD) approach[19–21] with a decomposition threshold of 10^{-4} a. u., and the second-order scalar-relativistic Douglas–Kroll–Hess one-electron Hamiltonian[22–24] was used to account for scalar relativistic effects.

The DMRG-SCF calculations have been performed in a state-average manner, averaging separately over the different spin states, i. e. as two separate state-average calculations averaging over 16 singlet and 10 triplet states, respectively. Out of these states, only 10 lowest singlet and 9 triplet states were considered due to intruder state problems with higher excited states and since higher-lying excited states are not relevant for the excitation near the absorption maximum anyway. Spin-orbit couplings were calculated with the SO-MPSSI[25,26] approach. All multiconfigurational calculations were performed with the OpenMOLCAS[27] program package and its interface to the QCMaquis DMRG program.[28]

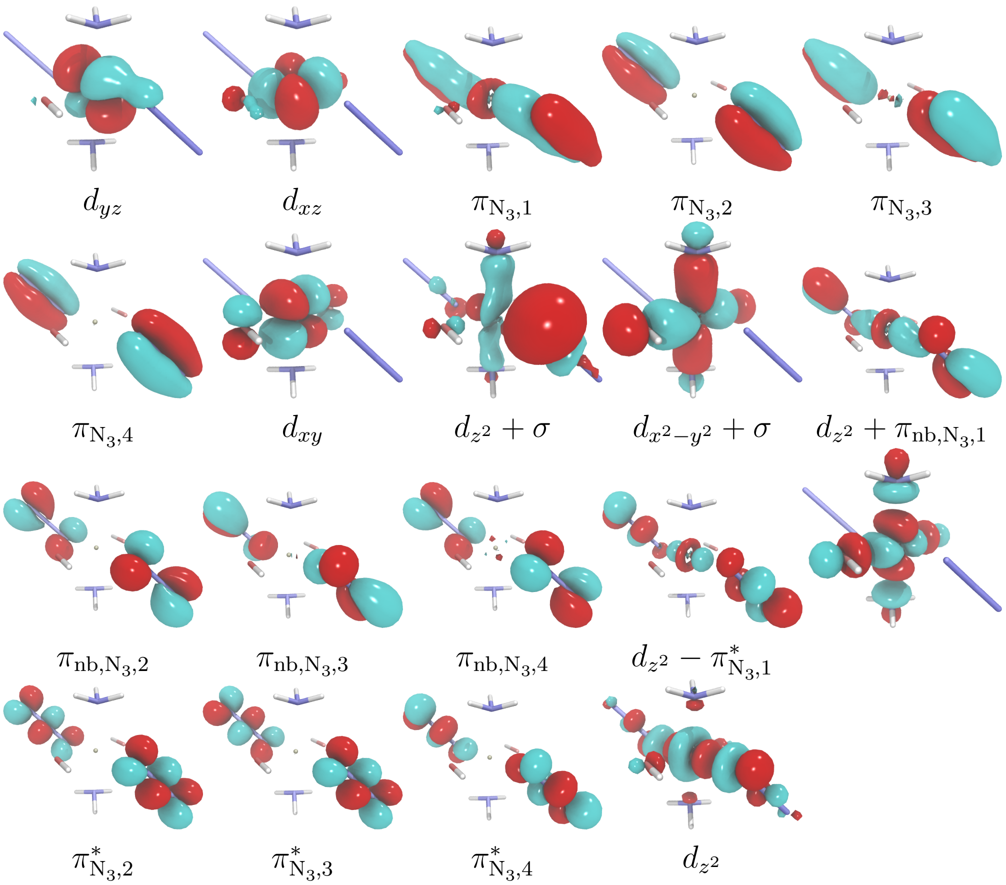

Figure S1: Active orbitals employed for DMRG-SCF calculations of the Pt complex discussed in this work. The plus or minus signs in the orbital description denote bonding and antibonding interaction of the orbitals.

Spin-free excitation energies

Table S1: Spin-free excitation energies, oscillator strengths and characters of the lowest singlet and triplet excited states of complex 1. The excited state characters are obtained from the natural transition orbitals (NTOs) for each state: orbital contributions in parentheses denote a small contribution, plus or minus signs denote bonding or antibonding interaction, respectively. The bright states are highlighted in bold.

State	ΔE/eV	f	Character
T_1	3.09		$(d_{xz}-)\pi_{nb,N_3,4} \rightarrow d_{z^2} - \pi_{nb,N_3,1}$
S_1	3.30	6.51×10^{-5}	$d_{xz} - \pi_{nb,N_3,4} \rightarrow d_{z^2} - \pi_{nb,N_3,1}$
T_2	3.94		$\pi_{nb,N_3,3} \rightarrow d_{z^2} - \pi_{nb,N_3,1}$
T_3	3.94		$d_{xy} \rightarrow d_{x^2-y^2}$
**S_2**	**4.01**	3.60×10^{-3}	$\pi_{nb,N_3,2} \rightarrow d_{z^2} - \pi_{nb,N_3,1}$
T_4	4.02		$d_{xy} \rightarrow d_{x^2-y^2}$
T_5	4.03		$\pi_{nb,N_3,3} \rightarrow d_{z^2} - \pi_{nb,N_3,1}$
S_3	4.41	0	$d_{xz} - \pi_{nb,N_3,4} \rightarrow d_{x^2-y^2}$
T_6	4.48		$d_{xy} \rightarrow d_{z^2} - \pi_{nb,N_3,1}$
S_4	4.65	1.84×10^{-5}	$d_{xy} \rightarrow d_{x^2-y^2}$
T_7	4.93		$d_{xy} \rightarrow d_{z^2}$
**S_5**	**4.93**	1.12	$d_{z^2} + \pi_{\mathbf{nb,N_3},3} \rightarrow d_{z^2} - \pi_{\mathbf{nb,N_3},1}$
T_8	5.02		$d_{xy} \rightarrow d_{x^2-y^2}$
S_6	5.30	0.0322	$d_{xz} \rightarrow d_{z^2} - \pi_{nb,N_3,1}$
T_9	5.39		$\pi_{nb,N_3,4} \rightarrow \pi^*_{N_3,2}$
S_7	5.46	0.172	$\pi_{nb,N_3,3} \rightarrow d_{x^2-y^2}$
S_8	5.49	0.0302	$\pi_{nb,N_3,2} \rightarrow d_{x^2-y^2}$
S_9	5.73	4.67×10^{-3}	$d_{xz} \rightarrow d_{z^2}$
S_{10}	5.83	1.44×10^{-4}	$d_{xy} \rightarrow d_{z^2}$

Spin-orbit states

Table S2: Excitation energies, oscillator strength and spin-free contributions over 5% of the lowest 37 spin-orbit (SO) excited states.

SO state	ΔE/eV	f	Spin-free contributions
1	0.00		95% S_0 + 2% T_3
2	3.27		96% T_1
3	3.28		97% T_1
4	3.31		97% T_1
5	3.49		96% S_1
6	3.86		47% T_3 + 44% T_5
7	3.92		54% T_3 + 42% T_5
8	4.03		79% T_3 + 10% S_3
9	4.08		78% T_5 + 12% S_5
10	4.16	3.13×10^{-4}	90% T_2 + 10% S_2
11	4.17	3.57×10^{-4}	96% T_2
12	4.17	9.31×10^{-4}	96% T_2
**13**	**4.24**	3.99×10^{-3}	**60% S_2 + 32% T_4 + 8% T_2**
**14**	**4.26**	1.14×10^{-3}	**96% T_4**
15	4.26	2.62×10^{-4}	96% T_4
16	4.26	7.37×10^{-4}	68% T_4 + 29% S_2
17	4.46		56% T_5 + 42% T_3
18	4.53		47% T_5 + 31% T_3 + 17% T_6
19	4.64		42% T_6 + 37% S_3 + 12% T_7
20	4.69		42% T_6 + 39% S_3 + 12% T_3
21	4.70	6.94×10^{-5}	72% T_6 + 9% T_7 + 8% T_3 + 5% S_3
22	4.77	6.05×10^{-4}	56% T_6 + 14% T_5 + 12% S_5 + 7% T_3
23	4.96	2.29×10^{-4}	58% S_5 + 17% T_6 + 10% T_8 + 10%T_5
**24**	**5.10**	4.60×10^{-2}	**72% T_7 + 11% S_6 + 7% S_4**
**25**	**5.16**	1.05	**90% S_4 + 6% T_7**
26	5.17	2.86×10^{-2}	54% T_7 + 20% T_8 + 14% T_6 + 5% S_3
27	5.19	1.66×10^{-3}	63% T_7 + 17% T_8 + 15% T_6
28	5.36	3.37×10^{-3}	79% T_8 + 12% S_5
29	5.38	1.24×10^{-4}	68% T_8 + 26% T_7
30	5.42	5.38×10^{-4}	69% T_8 + 19% T_7
31	5.58	1.13×10^{-2}	56% T_9 + 32% S_6 + 7% T_7
32	5.63		96% T_9
33	5.63	1.30×10^{-4}	96% T_9
34	5.68	2.25×10^{-2}	37% S_7 + 33% S_6 + 22% T_9
35	5.69	1.70×10^{-1}	62% S_7 + 16% S_6 + 15% T_9
36	5.72	1.64×10^{-2}	91% S_8 + 6% T_9
37	6.03	3.86×10^{-3}	93% S_9

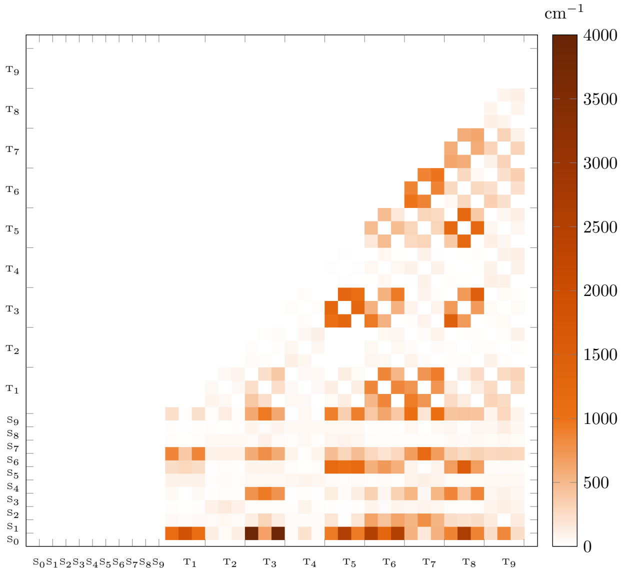

(a) equilibrium structure, $r = 2.077$ Å

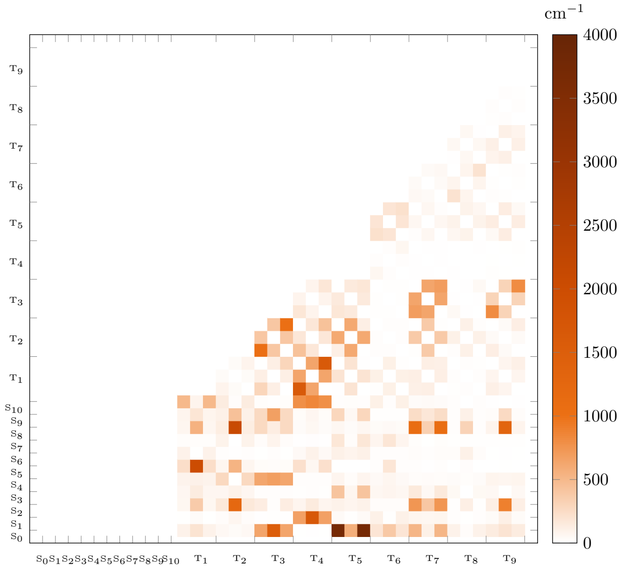

(b) $r = 2.677$ Å

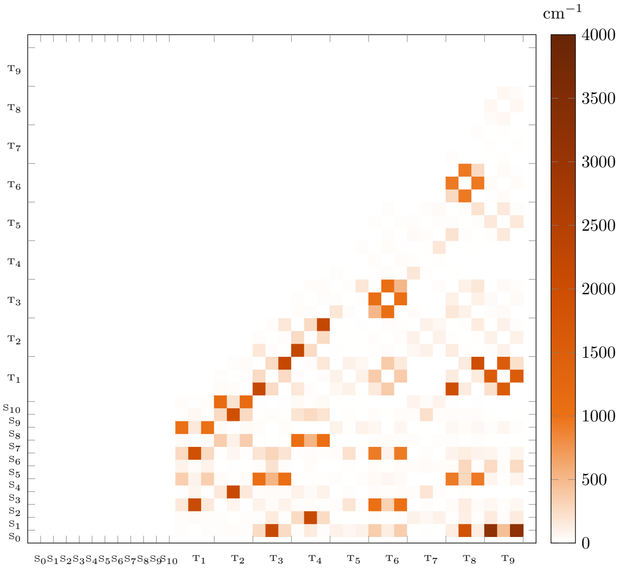

(c) $r = 3.877$ Å

Figure S2: Spin-orbit couplings at the equilibrium structure and extended Pt–N bond lengths. Only the lower triangle of the symmetric SOC matrix is shown.

DMRG-SCF excitation energies for $m = 500$ and $m = 1000$

Table S3: Absolute electronic and excitation energies for the singlet states of complex 1 at the equilibrium structure calculated with DMRG-SCF with $m = 500$ and $m = 1000$.

	$m = 500$		$m = 1000$		
State	E/a. u.	ΔE/eV	E/a. u.	ΔE/eV	$\lvert\Delta\Delta E\rvert$/eV
S_0	−18986.628352	0.000	−18986.629518	0.000	
S_1	−18986.507117	3.299	−18986.510878	3.228	0.071
S_2	−18986.480866	4.013	−18986.484218	3.954	0.059
S_3	−18986.466274	4.410	−18986.470670	4.323	0.088
S_4	−18986.457361	4.653	−18986.459081	4.638	0.015
S_5	−18986.447163	4.931	−18986.450503	4.871	0.059
S_6	−18986.433551	5.301	−18986.436647	5.248	0.053
S_7	−18986.427799	5.457	−18986.431869	5.378	0.079
S_8	−18986.426537	5.492	−18986.430339	5.420	0.072
S_9	−18986.417926	5.726	−18986.422526	5.633	0.093
S_{10}	−18986.413956	5.834	−18986.415890	5.813	0.021

Table S3 contains electronic energies for the first 11 singlet states of complex 1 at the equilibrium structure calculated with DMRG-SCF employing m values of 500 and 1000. While the electronic energies between the respective states differ of up to 5×10^{-3} a. u., the absolute difference between excitation energies (i. e. energy differences of the excited states to the respective ground state) is smaller than 0.1 eV for all states.

DMRG-NEVPT2 excitation energies

Table S4: DMRG-QD-NEVPT2 absolute electronic and excitation energies for the three lowest singlet states of complex 1 at the equilibrium structure.

State	E/a. u.	ΔE/eV
S_0	−18989.3046691	0.000
S_1	−18989.1941447	3.008
S_2	−18989.1656567	3.783

Table S4 shows the energies of three lowest singlet states of complex 1 at the equilibrium structure calculated with DMRG-QD-NEVPT2. Compared to DMRG-SCF results, the relative energy of S_1 is red-shifted by 0.30 eV and that of S_2 by 0.23 eV. We assume that a similar shift should be expected also for higher excited singlet states, and thus the inclusion of dynamic correlation should improve the agreement with the experiment by a similar amount.

Equilibrium structure coordinates

Table S5: XYZ coordinates of the equilibrium structure of complex 1

```
Pt 3.1357298 1.8682428 1.6662881
N 2.9910409 1.6496287 3.7011222
O 1.9031656 3.4633441 1.9204379
H 1.0038970 3.1049332 1.8144034
N 3.2790288 2.0879307 -0.3684170
H 3.7293402 1.2252149 -0.7033577
H 3.8853642 2.8922795 -0.5588309
H 2.3744459 2.2241222 -0.8258051
O 4.3669353 0.2722271 1.4124283
H 5.2667683 0.6299688 1.5156418
N 4.8088200 3.0949075 1.7712299
N 4.8126940 3.8902636 2.7003822
N 4.8893643 4.6481258 3.5687091
N 1.4620152 0.6430291 1.5589736
N 1.4690748 -0.1688260 0.6439364
N 1.4013038 -0.9422370 -0.2111235
H 2.3958750 0.8372778 3.8925206
H 2.5271984 2.5073194 4.0308875
H 3.8957385 1.5292177 4.1626831
```

References

(1) Perdew, J. P. Density-Functional Approximation for the Correlation Energy of the Inhomogeneous Electron Gas. *Phys. Rev. B* **1986**, *33*, 8822–8824.

(2) Becke, A. D. B3 Functional: Density-Functional Thermochemistry. III. The Role of Exact Exchange. *J. Chem. Phys.* **1993**, *98*, 5648–5652.

(3) Schäfer, A.; Horn, H.; Ahlrichs, R. Fully Optimized Contracted Gaussian Basis Sets for Atoms Li to Kr. *J. Chem. Phys.* **1992**, *97*, 2571–2577.

(4) Eichkorn, K.; Weigend, F.; Treutler, O.; Ahlrichs, R. Auxiliary Basis Sets for Main Row Atoms and Transition Metals and Their Use to Approximate Coulomb Potentials. *Theor. Chem. Acc.* **1997**, *97*, 119–124.

(5) Weigend, F.; Ahlrichs, R. Balanced Basis Sets of Split Valence, Triple Zeta Valence and Quadruple Zeta Valence Quality for H to Rn: Design and Assessment of Accuracy. *Phys. Chem. Chem. Phys.* **2005**, *7*, 3297–3305.

(6) Grimme, S.; Antony, J.; Ehrlich, S.; Krieg, H. A Consistent and Accurate Ab Initio Parametrization of Density Functional Dispersion Correction (DFT-D) for the 94 Elements H-Pu. *J. Chem. Phys.* **2010**, *132*, 154104.

(7) Pierloot, K. Transition Metals Compounds: Outstanding Challenges for Multiconfigurational Methods. *Int. J. Quantum Chem.* **2011**, *111*, 3291–3301.

(8) Pierloot, K.; Phung, Q. M.; Domingo, A. Spin State Energetics in First-Row Transition Metal Complexes: Contribution of (3s3p) Correlation and Its Description by Second-Order Perturbation Theory. *J. Chem. Theory Comput.* **2017**, *13*, 537–553.

(9) Sokolov, A. Y.; Schaefer III, H. F. Ground and Excited State Properties of Photoactive Platinum(IV) Diazido Complexes: Theoretical Considerations. *Dalton Trans.* **2011**, *40*, 7571–7582.

(10) Andrae, D.; Häußermann, U.; Dolg, M.; Stoll, H.; Preuß, H. Energy-Adjusted Ab Initio Pseudopotentials for the Second and Third Row Transition Elements. *Theoret. Chim. Acta* **1990**, *77*, 123–141.

(11) TURBOMOLE V7.0 2015, a development of University of Karlsruhe and Forschungszentrum Karlsruhe GmbH, 1989-2007, TURBOMOLE GmbH, since 2007; available from `http://www.turbomole.com`.

(12) Ghosh, D.; Hachmann, J.; Yanai, T.; Chan, G. K.-L. Orbital Optimization in the Density Matrix Renormalization Group, with Applications to Polyenes and β-Carotene. *J. Chem. Phys.* **2008**, *128*, 144117.

(13) Zgid, D.; Nooijen, M. The Density Matrix Renormalization Group Self-Consistent Field Method: Orbital Optimization with the Density Matrix Renormalization Group Method in the Active Space. *J. Chem. Phys.* **2008**, *128*, 144116.

(14) Angeli, C.; Cimiraglia, R.; Malrieu, J.-P. N-Electron Valence State Perturbation Theory: A Fast Implementation of the Strongly Contracted Variant. *Chem. Phys. Lett.* **2001**, *350*, 297–305.

(15) Angeli, C.; Cimiraglia, R.; Malrieu, J.-P. N-Electron Valence State Perturbation Theory: A Spinless Formulation and an Efficient Implementation of the Strongly Contracted and of the Partially Contracted Variants. *J. Chem. Phys.* **2002**, *117*, 9138–9153.

(16) Angeli, C.; Borini, S.; Cestari, M.; Cimiraglia, R. A Quasidegenerate Formulation of the Second Order N-Electron Valence State Perturbation Theory Approach. *J. Chem. Phys.* **2004**, *121*, 4043–4049.

(17) Freitag, L.; Knecht, S.; Angeli, C.; Reiher, M. Multireference Perturbation Theory with Cholesky Decomposition for the Density Matrix Renormalization Group. *J. Chem. Theory Comput.* **2017**, *13*, 451–459.

(18) Roos, B. O.; Lindh, R.; Malmqvist, P.-A.; Veryazov, V.; Widmark, P.-O. New Relativistic ANO Basis Sets for Transition Metal Atoms. *J. Phys. Chem. A* **2005**, *109*, 6575–6579.

(19) Aquilante, F.; Pedersen, T. B.; Lindh, R.; Roos, B. O.; Sánchez de Merás, A.; Koch, H. Accurate Ab Initio Density Fitting for Multiconfigurational Self-Consistent Field Methods. *J. Chem. Phys.* **2008**, *129*, 024113.

(20) Aquilante, F.; Gagliardi, L.; Pedersen, T. B.; Lindh, R. Atomic Cholesky Decompositions: A Route to Unbiased Auxiliary Basis Sets for Density Fitting Approximation with Tunable Accuracy and Efficiency. *J. Chem. Phys.* **2009**, *130*, 154107.

(21) Pedersen, T.; Aquilante, F.; Lindh, R. Density Fitting with Auxiliary Basis Sets from Cholesky Decompositions. *Theor. Chem. Acc.* **2009**, *124*, 1–10.

(22) Hess, B. A. Relativistic Electronic-Structure Calculations Employing a Two-Component No-Pair Formalism with External-Field Projection Operators. *Phys. Rev. A* **1986**, *33*, 3742–3748.

(23) Wolf, A.; Reiher, M.; Hess, B. A. The Generalized Douglas–Kroll Transformation. *J. Chem. Phys.* **2002**, *117*, 9215–9226.

(24) Reiher, M.; Wolf, A. Exact Decoupling of the Dirac Hamiltonian. II. The Generalized Douglas–Kroll–Hess Transformation up to Arbitrary Order. *J. Chem. Phys.* **2004**, *121*, 10945–10956.

(25) Malmqvist, P. A.; Roos, B. O.; Schimmelpfennig, B. The Restricted Active Space (RAS) State Interaction Approach with Spin–Orbit Coupling. *Chem. Phys. Lett.* **2002**, *357*, 230–240.

(26) Knecht, S.; Keller, S.; Autschbach, J.; Reiher, M. A Nonorthogonal State-Interaction Approach for Matrix Product State Wave Functions. *J. Chem. Theory Comput.* **2016**, *12*, 5881–5894.

(27) Fdez. Galván, I.; Vacher, M.; Alavi, A.; Angeli, C.; Aquilante, F.; Autschbach, J.;

Bao, J. J.; Bokarev, S. I.; Bogdanov, N. A.; Carlson, R. K. et al. OpenMolcas: From Source Code to Insight. *J. Chem. Theory Comput.* **2019**, *15*, 5925–5964.

(28) Keller, S.; Dolfi, M.; Troyer, M.; Reiher, M. An Efficient Matrix Product Operator Representation of the Quantum Chemical Hamiltonian. *J. Chem. Phys.* **2015**, *143*, 244118.
